# Construction of an Onion (*Allium cepa* L.) Genetic Linkage Map Using Genotyping-by-Sequencing Analysis with a Reference Gene Set and Identification of QTLs Controlling Anthocyanin Synthesis and Content

**DOI:** 10.3390/plants9050616

**Published:** 2020-05-12

**Authors:** Yousoo Choi, Sunggil Kim, Jundae Lee

**Affiliations:** 1 Department of Horticulture, Institute of Agricultural Science & Technology, Jeonbuk National University, Jeonju 54896, Korea; youso1127@naver.com; 2 National Agrobiodiversity Center, National Institute of Agricultural Sciences, Rural Development Administration, Jeonju 54874, Korea; 3 Department of Plant Biotechnology, Biotechnology Research Institute, Chonnam National University, Gwangju 61186, Korea; dronion@jnu.ac.kr

**Keywords:** anthocyanin, bulb color, GBS, HRM, QTL, transcriptome

## Abstract

Anthocyanins, the pigmented flavonoids responsible for red and blue colors in horticultural products, promote human health by preventing cancers and lowering the risk of cardiovascular disease. Red onions contain several cyanidin- and peonidin-based anthocyanins. In this study, we constructed a single-nucleotide polymorphism (SNP)-based genetic linkage map in an F_2_ segregating population derived from a cross between the inbred line ‘SP3B’ (yellow bulb) and the doubled haploid line ‘H6′ (red bulb) to identify quantitative trait loci (QTLs) for total anthocyanin content of onion bulbs using a genotyping-by-sequencing (GBS) analysis based on a reference gene set. A total of 101.9 Gbp of raw sequences were generated using an Illumina HiSeq 2500 system and a total of 1625 SNP loci were identified with the criteria of three minimum depths, lower than 30% missing rate, and more than 5% minor allele frequency. As a result, an onion genetic linkage map consisting of 319 GBS-based SNP loci and 34 high-resolution melting (HRM) markers was constructed with eight linkage groups and a total genetic distance of 881.4 cM. In addition, the linkage groups were assigned to corresponding chromosomes by comparison with the reference genetic map OH1×5225 through marker development based on common transcripts. The analysis revealed one major QTL, *qAS7.1*, for anthocyanin synthesis and two significant QTLs, *qAC4.1* and *qAC4.2*, for anthocyanin content. The QTL *qAS7.1*, located on chromosome 7 with a phenotypic variation of 87.61%, may be a dihydroflavonol 4-reductase (*DFR*) gene that determines whether the bulb color is red or yellow. The QTLs *qAC4.1* and *qAC4.2* are separately positioned on chromosome 4 with *R*^2^ values of 19.43% and 26.28%, respectively. This map and QTL information will contribute to marker development and breeding for high anthocyanin content in bulb onion.

## 1. Introduction

Bulb onion (*Allium cepa* L.; 2n = 2x = 16) is an economically and nutritionally important vegetable crop worldwide [1]. The health benefits of onion are due to several functional compounds, including anthocyanins (mainly in red/purple onions), flavanols such as quercetin (mainly in yellow/brown onions), and alk(en)yl cysteine sulphoxides (ACSOs) [2]. Anthocyanins, type of flavonoid, are water-soluble vacuolar pigments that confer red, blue, and purple colors in horticultural products depending on the pH [3]. Red onions contain four major cyanidin-based anthocyanins; cyanidin 3-glucoside (Cy 3-Glc), cyanidin 3-laminaribioside (Cy 3-Lam), cyanidin 3-malonylglucoside (Cy 3-MaGlc), and cyanidin 3-malonyllaminaribioside (Cy 3-MaLam) [4], and two minor peonidin derivatives, peonidin 3-glucoside and peonidin 3-malonylglucoside [5].

Inheritance of onion bulb colors appears in a complex pattern [6,7]. Previous inheritance studies have reported the presence of six major loci (*I*, *C*, *G*, *L*, *L2*, and *R*) that are responsible for bulb colors [1]. Particularly, the *R* locus and either the *L* or *L2* locus are complementarily involved in the control of yellow and red bulb colors [7,8], and the *R* and *L* loci were reported to correspond to the dihydroflavonol 4-reductase (*DFR*) and anthocyanidin synthase (*ANS*) genes, respectively [8]. The *DFR* and *ANS* genes were assigned to chromosomes 7 and 4, respectively; using two complete sets of shallot (*A. cepa*) alien monosomic addition lines [9]. Transcripts of the *DFR* gene were seen in red onions but were absent in yellow onions [10], and it was suggested that blockage of *DFR* transcription or translation results in a lack of anthocyanin production in yellow onion [11]. A total of 16 *DFR-A* alleles were identified, and the process for identification of the alleles was reported [12,13,14]. On the other hand, *ANS* was related to a pink color as well as anthocyanin production in onion [15,16,17,18]. However, the genetic inheritance of anthocyanin content in red onion is poorly understood.

Genomic and genetic studies of onion are difficult due to its huge genome size (16.3 Gbp), biennial life cycle, cross-pollinating nature, and high inbreeding depression [1]. For these reasons, the whole genome sequencing of the onion is not yet completed [19]. Despite the difficulties, several studies on genetic linkage mapping and marker development in the onion have been made. A low-density genetic map of the onion, including 14 random amplified polymorphic DNA (RAPD) and 110 restriction fragment length polymorphism (RFLP) markers, was first constructed using 58 F_3_ families derived from a single F_1_ plant from the cross of ‘Brigham Yellow Globe 15-23′ (BYG15-23) and ‘Alisa Craig 43′ (AC43) [20]. An interspecific genetic map of *A. roylei* × *A. cepa* was made using 692 amplified fragment length polymorphism (AFLP) markers [21]. In addition, the linkage groups were assigned to the chromosomes of *A. cepa* L. via monosomic addition lines [22]. A total of 13 markers, including two cleaved amplified polymorphic sequence (CAPS) and 11 single-strand conformation polymorphism (SSCP) markers, were developed from 128 expressed sequence tag (EST) sequences and positioned on the ‘BYG15-23′ × ’AC43′ map [23]. In the same population, 100 new genetic markers were developed from EST sequences and the ‘BYG15-23′ × ’AC43′ map consisting of 14 linkage groups encompassing 1907 cM was constructed [24]. A total of 37 simple sequence repeat (SSR) markers were developed to distinguish between 35 onion cultivars [25], and 56 EST-SSR and four genomic SSR markers were used for genetic diversity analysis of 89 inbred and open-pollinated bulb onions [26].

Next-generation sequencing (NGS) technologies have made it easier to identify a large number of single-nucleotide polymorphisms (SNPs) to develop SNP markers and to construct genetic linkage maps for plant genetics and breeding [27]. A total of 205 markers, including 11 indel, 90 CAPS, and 104 high-resolution melting (HRM) markers, have been developed from NGS data, and a framework linkage map of over 800 cM spanning all chromosomes was constructed in an F_2_ population from a cross between the two bulb onions ‘Nasik Red’ and ‘CUDH2150’ [28]. The 20 robust single copy SSR markers selected from 166 SSRs were used for the estimation of genetic diversity within and among 24 bulb onion populations [29]. A total of 597 SNPs identified from cDNA libraries between the bulb onions ‘OH1′ and ‘5225′ were positioned on a genetic map consisting of ten linkage groups, and the map was compared with the ‘BYG1523′ × ‘AC43′ map using 223 common SNPs [30]. A total of 54,165 protein-coding genes among 165,179 assembled transcripts totaling 203 Mb were generated with de novo high-throughput RNA sequencing (RNA-Seq) analysis [31]. In addition, 35,505 isoforms, designated as draft reference transcripts (DRTs, version 1.0), were produced using long-read sequencing [32]. The 301 SNP markers based on kompetitive allele specific PCR (KASP) assays were developed using transcriptome sequencing, and two interspecific genetic maps between *A. roylei* and *A. fistulosum* and between *A. cepa* and *A. roylei* were constructed using the SNP markers [33]. From genotyping-by-sequencing (GBS), 175 SNPs and 57 from Fluidigm SNP assays were used for the construction of an onion genetic map, which consisted of eight linkage groups and covered a total length of 1339.5 cM [34]. A total of 1904 SNPs were discovered in 192 Korean short-day onion inbred lines using double digest restriction site-associated DNA sequencing (ddRAD-seq) [35].

In this study, we aimed to construct an onion genetic linkage map using GBS analysis with the previously reported reference gene set and without the reference whole-genome sequence and to identify quantitative trait loci (QTLs) controlling anthocyanin synthesis and content in an F_2_ population.

## 2. Results

### 2.1. SNP Detection and Genotyping Using GBS Analysis

GBS analysis was carried out with 96 F_2_ onion plants for SNP detection and genotype identification. In total, one billion raw reads and 101.9 Gbp of sequences were obtained using Illumina HiSeq 2500 paired-end sequencing (Table 1). The raw reads were classified into 96 groups (samples) using the barcode sequences. The average number of reads in each group was 10,075,947. Subsequently, the demultiplexed reads were trimmed by eliminating barcodes, adaptors, and low-quality sequences. The average length of trimmed reads per sample was 77.75 bp, and accounts for 86.2% of the total raw reads (Table 1). The trimmed reads were mapped on the reference gene set of bulb onion (Table 2) [31]. As a result, only 35.5% of the raw reads were mapped and the total number of mapped reads was 358,301,156 (Table 1). The average number of each mapped region was 16,718, and the average depth of each mapped region was 81.42 (Table 1). The average length of the mapped regions was 1,855,555 bp, which covered 0.9141% of the onion reference gene set (Table 1). Finally, a SNP matrix consisting of 96 samples and 8431 SNPs was generated (Table 1). After filtering with a minimum depth of three, less than 30% missing rate, and over 5% major allele frequency, 1625 SNPs were obtained (Table 1 and Table S1).

### 2.2. Development of HRM Markers for Comparative Mapping

**Figure 1:**
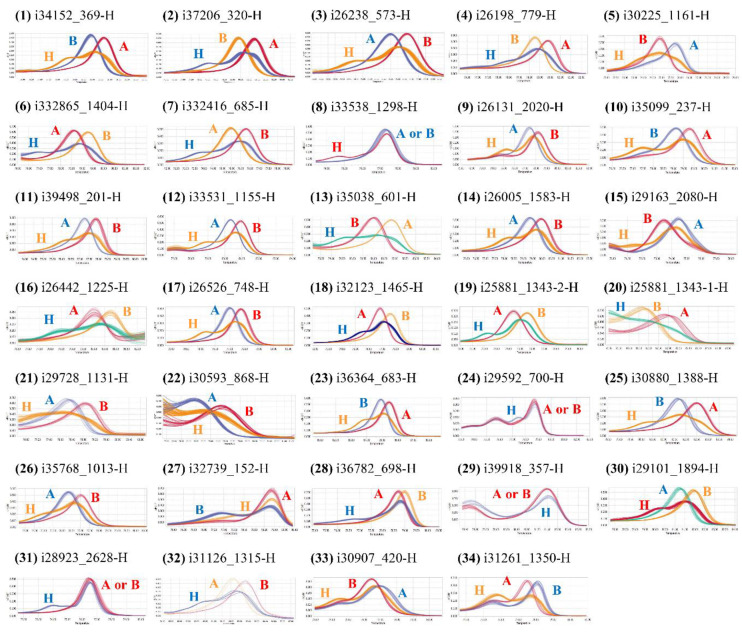
Melting curves of 34 high-resolution melting (HRM) markers developed in this study. A, marker type of female parent (SP3B); B, marker type of male parent (H6); H, marker type of heterozygote.

A total of 248 primer sets for HRM markers were designed based on common SNPs between the populations SP3B×H6 (in this study) and OH1×5225 [30]. Among them, only 34 were polymorphic (Figure 1). The HRM marker types were clearly separated into three groups: A (SP3B genotype), B (H6 genotype), and H (heterozygous genotype; Figure 1). The markers were positioned widely throughout the genome on chromosomes 1–8. Detailed marker information is listed in Table 3.

### 2.3. Construction of an Onion Genetic Linkage Map

**Figure 2:**
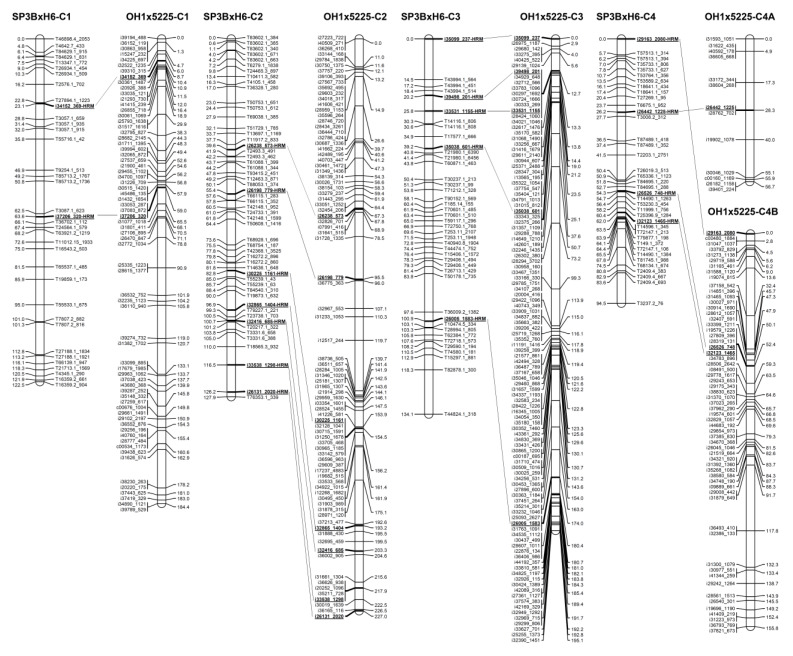

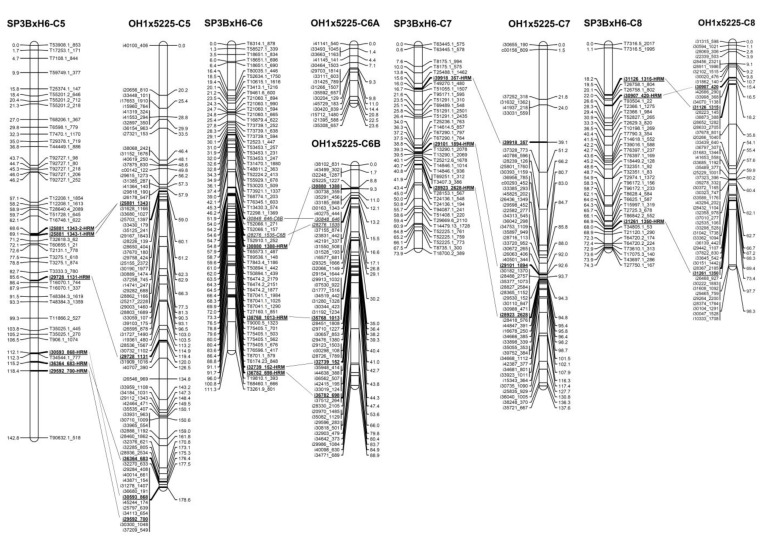
Comparison of two onion genetic linkage maps, the SP3B×H6 map constructed in this study and the OH1×5225 map developed by Duangjit et al. [30]. Bar left or right number, map position (centi Morgan, cM); bar left or right name, marker name; underline, common marker; -HRM in marker name, HRM marker; dotted line, connection between the same transcript-based markers.

An onion genetic linkage map consisting of 319 GBS-based SNPs and 34 HRM markers on eight chromosomes was constructed with a total genetic distance of 881.4 cM (Figure 2, Table 4 and Table S2). The number of markers on each chromosome ranged from 36 to 64 with an average of 44, and the average marker interval was 2.5 cM (Table 4). The shortest and longest chromosomes were 7 and 5, with genetic distances of 73.9 cM and 142.8 cM, respectively (Table 4).

### 2.4. Comparison of the SP3B×H6 and OH1×5225 Onion Genetic Linkage Maps

The SP3B×H6 genetic linkage map generated in this study was compared with the previously reported onion reference genetic map for OH1×5225 to assign each linkage group to the corresponding chromosome (Figure 2). First, through a BLAST search with the mapped transcript sequences, only seven common transcripts were identified; including three (i33531_1155, i35038_601, and i31357_1109) on chromosome 3, one (i37258_745) on chromosome 5, two (i30848_646 and i28276_1535) on chromosome 6B, and one (i31126_1315) on chromosome 8 (Figure 2). Second, a total of 34 HRM markers were developed using SNPs derived from common transcripts (Figure 1 and Table 3). These markers enabled the comparison between SP3B×H6 and OH1×5225 (Figure 2).

### 2.5. Identification of a Major QTL for Anthocyanin Synthesis of Onion Bulbs

**Figure 3:**
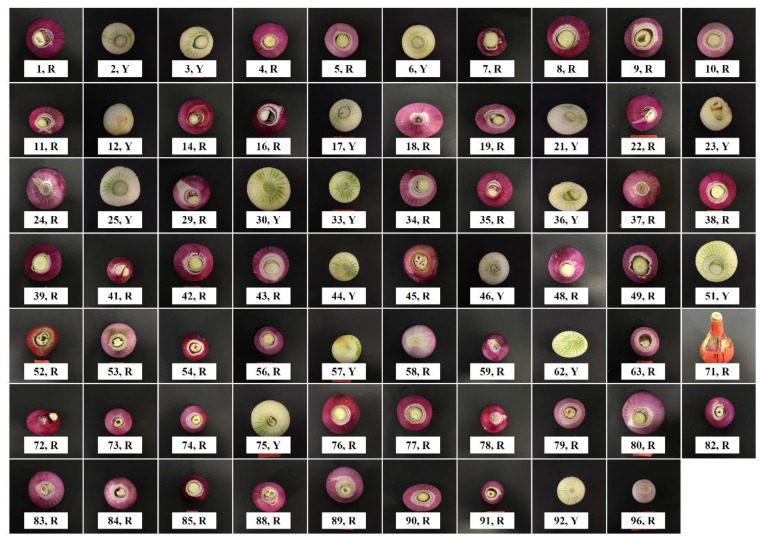
Bulb colors of 69 F_2_ individuals derived from a cross between *Allium cepa* ‘SP3B’ and ‘H6′. Numbers, No. of F_2_ individual; R, red color; Y, yellow color.

Only 69 bulbs were harvested from 96 F_2_ onion plants due to cultivation problems, which were segregated into 51 red bulbs and 18 yellow bulbs (Figure 3), fitting to the segregating ratio of 3:1 (red:yellow; Table 5). This shows that the red bulb color is caused by the expression of a single dominant gene and yellow bulb color results from homozygous recessive alleles of the gene. QTL analysis using these data revealed a major QTL, *qAS7.1*, for anthocyanin synthesis in the onion (Table 6). This QTL was identified at the 13.8 cM position on chromosome 7 with a logarithm of odds (LOD) score of 9.19 and a phenotypic variance of 87.61% (Table 6). The segregation of red and yellow bulbs was completely consistent with the genotype of the closest marker (T25488.1_1462) to *qAS7.1* (Figure 2 and Table S3). In the marker, the homozygous genotype of SP3B led to yellow bulbs, whereas the homozygous genotype of H6 or the heterozygous genotype caused red bulbs.

### 2.6. Identification of Two QTLs for Anthocyanin Content of Onion Bulbs

**Figure 4:**
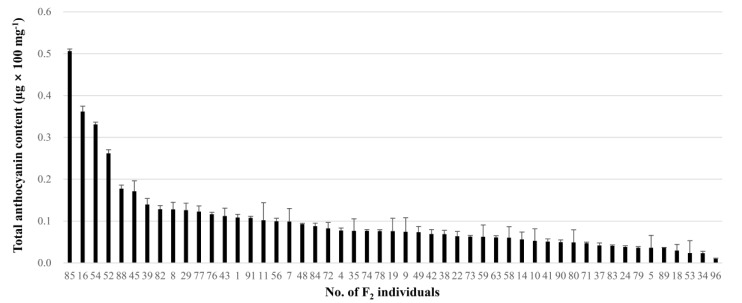
Total anthocyanin content of 51 red bulbs in the F_2_ population of SP3B×H6.

**Figure 5:**
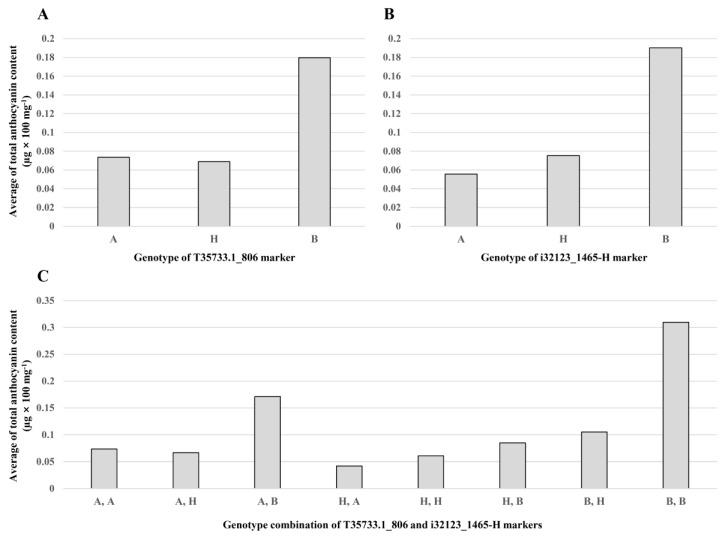
Comparison of the average total anthocyanin content according to genotypes for T35733.1_806 (**A**) and i32123_1465-H (**B**) markers and their combinational genotype (**C**). A, genotype of female parent (SP3B); B, genotype of male parent (H6); H, genotype of heterozygote.

Anthocyanin content of 51 red bulbs in the F_2_ population ranged from 0.0098 to 0.5061 µg × 100 mg^−1^ (Figure 4). The average anthocyanin content was 0.0994 µg × 100 mg^−1^, and the average standard deviation was 0.0133 µg × 100 mg^−1^. This continuous variation of anthocyanin content indicates that it is quantitatively controlled (Figure 4). Using this data, two significant QTLs, *qAC4.1* and *qAC4.2*, for anthocyanin content of onions were identified by a QTL analysis (Table 6). The QTLs were found at 7 and 62 cM on chromosome 4 with LOD scores of 3.26 and 3.03 and *R*^2^ values of 19.43% and 26.28%, respectively (Table 6). The negative additive effects on anthocyanin content were derived from the SP3B genotype and observed in both QTLs (Table 6). A GBS-based marker, T35733.1_806, and an HRM-based marker, i32133_1465-HRM, were the closest markers to *qAC4.1* and *qAC4.2*, respectively (Figure 2 and Table 6). For both markers, the homozygous paternal genotype (B) had the highest total anthocyanin content (0.18 µg × 100 mg^−1^ in T35733.1_806 and 0.19 µg × 100 mg^−1^ in T5789-1-C4; Figure 5A,B). The onion lines with both paternal alleles for the markers also showed the highest total anthocyanin content (0.31 µg × 100 mg^−1^; Figure 5C).

## 3. Discussion

The whole-genome sequence of the bulb onion is not yet made public. Nevertheless, we successfully constructed an onion genetic linkage map using a GBS analysis with the reference of transcriptome sequences (Figure 2). There have been a few reports on the onion genetic map construction using NGS technologies. Baldwin et al. [28] reported 195 molecular markers including 11 Indels, 90 CAPSs, and 104 HRMs derived from NGS data, and Duangjit et al. [30] generated 597 SNP markers from transcriptome sequences using the KASP platform. Jo et al. [34] developed an onion genetic map using reference-free GBS analysis, but only 175 SNPs were mapped. We mapped a total of 319 SNPs in this study, divided into eight linkage groups covering a genetic distance of 881.4 cM (Figure 2 and Table 4). This is the first paper that reports the construction of an onion genetic linkage map using GBS analysis with a reference transcriptome sequence. This method will be useful for onion genetic mapping until the whole genome sequence is released.

The onion genetic linkage map of SP3B×H6 was compared with the previously reported genetic map of OH1×5225 using common SNP markers (Figure 2) [30]. The 34 developed HRM markers were widely distributed throughout the genome (Figure 2 and Table 3). All the linkage groups of SP3B×H6 were assigned to the corresponding chromosomes of OH1×5225 (Figure 2). By doing so, we were able to compare the positions of the QTLs to those detected by Duangjit et al. [36].

Segregation analysis of red and yellow bulb colors revealed the presence of one dominant gene responsible for red color through anthocyanin synthesis (Figure 3 and Table 5). Additionally, QTL analysis for anthocyanin synthesis identified only one major QTL, *qAS7.1*, on chromosome 7 with a high *R*^2^ value of 87.61% (Table 6). In previous studies, El-Shafie and Davis [7] proposed that red bulbs are conditioned by dominant alleles at the *R* and *L* loci, and Kim et al. [10,16] suggested that the *R* locus is *DFR* and the *L* locus is *ANS*. These genes were proven to be complimentarily involved in the control of red and yellow bulb colors using molecular markers [10,11,12,14,16,18]. In addition, hybridization analysis using alien monosomic addition lines suggested that the *DFR* and *ANS* genes are located on onion chromosomes 7 and 4, respectively [9]. In this study, *qAS7.1*-linked marker (T25488.1_1462) cosegregated with bulb colors (red and yellow; Table S3). These results imply that the strongest candidate gene for the QTL *qAS7.1* is *DFR*. However, *DFR* was not possible to be positioned in this map because a marker for the gene was not developed. We suggest further research on marker development for *DFR* gene to clarify the assumption.

The distribution of anthocyanin content in F_2_ onion bulbs suggests the trait is quantitatively controlled (Figure 4). QTL analysis for anthocyanin content of onion bulbs revealed two significant QTLs, *qAC4.1* and *qAC4.2*, on chromosome 4 (Table 6). In a previous study, Duangjit et al. [36] identified four QTLs on chromosomes 1, 4, and 8 for anthocyanin concentration and intensity of the red bulb color using segregating haploid progenies of the onion derived from a cross between OH1 (yellow) and 5225 (red). The QTL on chromosome 4 was closely linked to the three markers c00160_1169, i26182_1158, and i39401_224, which were positioned between 55.9 and 56.7 cM (Figure 2) [30]. It might also correspond to the QTL *qAC4.1* since they are located in a similar position (Figure 2). In this study, we additionally found the QTL *qAC4.2*, which was not detected in the previous study, and no QTLs identified on chromosomes 1 and 8 (Table 6). The *L* locus was proposed to encode the *ANS* gene, which is located on onion chromosome 4 [9,16], and Khar et al. [8] reported an additional locus (*L2*) on chromosome 4 linked to the *L* locus that also interacts with the *R* locus to regulate red bulb color. Therefore, the QTLs *qAC4.1* and *qAC4.2* need to be compared with the *L* and *L2* loci.

The two markers (T35733.1_806 and i32123_1465-H) were closely linked to the QTLs (*qAC4.1* and *qAC4.2*), respectively (Table 6). The genotypic analysis of the markers showed that the homozygous paternal genotype (B) had the highest anthocyanin content and heterozygous genotype (H) was similar to the homozygous maternal genotype (A; Figure 5A,B). These results suggest that both QTLs are recessive genes. In addition, simultaneous homozygous paternal genotype markers (B and B) showed exclusively high anthocyanin content (Figure 5C). This result means that the two QTLs have complementary interaction. Hence, these markers are believed to be very useful for marker-assisted selection for high anthocyanin content in onion breeding.

## 4. Materials and Methods

### 4.1. Plant Materials

An F_2_ segregating population consisting of 96 individuals was used to construct an onion genetic map and identify QTLs for anthocyanin synthesis and content. The population was generated by self-pollination of an F_1_ hybrid crossed between an inbred onion line with yellow bulb (SP3B) as a maternal line and a short-day type doubled haploid (DH) onion line with red bulb (H6) as a paternal line. The plants were cultivated in the open farm fields of Chonnam National University (Gwangju, South Korea) from October 2017 to June 2018.

### 4.2. Phenotyping of Bulb Color

Phenotypes of anthocyanin-presence and -absence were discriminated by visually observing bulb color; red and yellow bulbs indicate anthocyanin presence and absence, respectively. These phenotypic data were used to identify the position(s) of the gene(s) controlling anthocyanin synthesis.

### 4.3. Assessment of Anthocyanin Content

Total anthocyanin content of the F_2_ onion bulbs was assessed according to the method described by Shin et al. [37]. Anthocyanin extraction was performed as follows: the twelfth piece of bulb per each sample was crumbled in a mortar with liquid nitrogen. Of the bulb powder 100 mg was placed in a 2.0 mL microcentrifuge tube with 600 μL of extraction buffer (methanol containing 1% HCl), incubated for 6 h at 4 °C in dark, and then centrifuged at 4 °C for 5 min at 13,000 rpm using a centrifuge (Hanil Scientific Inc., Gimpo, Korea). A 600-μL aliquot of the supernatant was transferred to a new 1.5-mL microcentrifuge tube, and 200 μL of distilled water and 200 μL of chloroform:isoamyl alcohol (24:1) were added. The mixture was centrifuged for 5 min at 13,000 rpm at 4 °C, and 750 μL was then transferred to a new 1.5-mL microcentrifuge tube. An aliquot of 300 μL was transferred to a new 96-well microplate, and absorbances were measured at 530 nm and 657 nm using an Epoch microplate spectrophotometer (BioTek Instruments, Inc., Winooski, VT, USA). The degree of total anthocyanin content was determined by calculating the following function: total anthocyanin content = (A_530nm_) − 0.25 × (A_657nm_) [38]. Total anthocyanin content was assessed five times per sample and averaged.

### 4.4. DNA Extraction

Genomic DNA was extracted from young leaves of each F_2_ individual according to the method described by Lee et al. [39]. The DNA was dissolved in 100 μL of distilled water and treated with 0.1 μL of 10 mg·mL^−1^ RNase solution (Bio Basic Canada Inc., Ontario, Canada). The DNA concentration was measured using a BioDrop LITE (BioDrop UK Ltd., Cambridge, UK).

### 4.5. Genotyping-by-Sequencing Analysis

Genomic DNAs from 96 F_2_ individuals were used to construct the library for GBS analysis. The GBS library was constructed according to the method by Eun et al. [40] with the exception of double digestion with the two restriction enzymes *Pst*I and *Msp*I. The pooled GBS library was sequenced using a HiSeq 2500 (Illumina, Inc., San Diego, CA, USA) using the paired-end read method. The raw sequences were demultiplexed into 96 samples and the demultiplexed sequences were trimmed by removing the barcode, the adapter, and low-quality sequences. The cleaned sequences were aligned to the onion reference gene set consisting of 165,197 assembled contigs (Table 2) [31] using the Burrows–Wheeler alignment (BWA) program version 0.6.1-r104 [41]. Raw SNP detection, consensus sequences extraction, and SNP matrix generation were performed according to the method by Eun et al. [40].

### 4.6. High-Resolution Melting Analysis

HRM analysis was conducted according to the method described by Jeong et al. [42] using a LightCycler^®^ Real-Time PCR (Roche, Basel, Switzerland). The melting curve was analyzed with High-Resolution Melt software version 1.1 (Roche), and the genotypes were classified into three groups: A (SP3B marker type), B (H6 marker type), and H (heterozygous marker type). The newly developed polymorphic HRM markers were added to the SP3B×H6 map and compared with the OH1×5225 genetic linkage map [30].

### 4.7. Genetic Linkage Mapping

Genetic linkage maps were constructed using the JoinMap version 4.1 (Kyazma B.V., Wageningen, The Netherlands). Only SNPs fitting with the 1:2:1 ratio of the χ^2^-test were used (Table S2). A logarithm of odds (LOD) score of 3.0 was regarded as the threshold to determine the significant linkage between markers. Genetic map distances (cM) were calculated by the Kosambi mapping function [43]. The final linkage map was created using the MapChart version 2.1 software [44].

### 4.8. Assignment of Linkage Groups to Onion Chromosomes

Common transcripts were used to assign the linkage groups to onion chromosomes. The transcripts of the reference gene set [31] were compared to those of the standard genetic linkage map OH1×5225 [30]. In addition, a total of 248 primer sets for HRM markers were newly designed, which were selected from the SNPs derived from common transcripts between the reference gene set and the previous map OH1×5225.

### 4.9. QTL Analysis

QTL analysis was conducted using windows QTL Cartographer version 2.5 program [45] with the composite interval mapping (CIM) method. The LOD threshold for significance level (*p* = 0.05) was estimated with a 1000 times permutation test. QTL analysis was carried out using two phenotypic data: anthocyanin synthesis (AS) and anthocyanin content (AC). Anthocyanin synthesis indicates the presence or absence of anthocyanin, while anthocyanin content refers to high or low amounts of anthocyanin.

## 5. Conclusions

In summary, we performed GBS and HRM analyses on 96 F_2_ onion plants and constructed a genetic linkage map with 319 SNPs and 34 HRM markers, consisting of eight linkage groups and covering 881.4 cM with an average marker interval of 2.5 cM. Through QTL analysis, we identified a major QTL, *qAS7.1*, for anthocyanin synthesis and two significant QTLs, *qAC4.1* and *qAC4.2*, for anthocyanin content in the onion. In conclusion, the map information of the transcripts and markers will contribute to complete the onion whole-genome sequencing, and the QTL information for anthocyanin synthesis and content will be useful for molecular marker development for marker-assisted selection (MAS). This will help to facilitate the breeding of bulb onions with higher anthocyanin content.

## Figures and Tables

**Table 1:** Summary of genotyping-by-sequencing data generated by using transcriptome sequences as a reference.

Summary of Illumina Sequencing	Data
Number of plants for multiplexing	96
Total number of raw reads generated	1,008,750,538 (100%)
Total base number of raw reads (bp)	101,883,804,338 (101.9 Gbp)
Total number of demultiplexed reads	967,290,922 (95.9%)
Total number of trimmed reads	869,413,090 (86.2%)
Total number of mapped reads	358,301,156 (35.5%)
Total number of mapped regions	1,604,901
Average depth of mapped region	81.42
Total length of mapped regions (bp)	1,855,555 (1.9 Mbp)
Total length of the reference gene set (bp)	202,991,716 (203.0 Mbp)
Coverage of the reference gene set	0.9141%
Total number of SNPs detected	8431
Total number of SNPs filtered	1625

**Table 2:** Summary of transcript-assembled contigs used as an onion reference reported by Kim et al. [31].

Number of Assembled Contigs	Total Length (bp)	Minimum Length (bp)	Maximum Length (bp)	Average Length (bp)	N_50_ Length (bp)
165,179	202,991,716	200	16,504	1228	1756

**Table 3:** List of HRM markers developed in this study.

No.	SP3B×H6 Map							OH1×5225 Map ^z^		Transcript ID ^y^
	Chr. No. ^x^	Position (cM)	Marker Name	Forward Primer	Reverse Primer	H6	SP3B	Chr. No. ^x^	Position (cM)	AC.Combine.Assembly.v.1.0
1	1	23.1	i34152_369-HRM	TCCACATATCTCATATTGCGCTCA	CTTTGGCTTAACTTACCCGATTAC	G	A	1	8.7	AC.Combine.Locus_5700
2	1	63.6	i37206_320-HRM	CCGGTTGTGGTTGGTCGAA	ACAAGTTAGTGGCACGTTACAAAA	G	T	1	59.0	AC.Combine.Locus_9298
3	2	39.6	i26238_573-HRM	ACAAACCTTATGCAGATACACTCA	GCAACATCAAAAGCTCCCCATC	T	C	2	67.3	AC.Combine.Locus_14118
4	2	55.4	i26198_779-HRM	TTCTATTACCGGAGCTGTAGTTGG	CAAATGCAATATCTCCAAGGGCTT	G	A	2	95.5	AC.Combine.Locus_9799
5	2	82.8	i30225_1161-HRM	GAAGGGACAGTTCAAGGTAGTAGG	TCTCAAATTCCTTCTCCAACTTCA	G	A	2	154.5	AC.Combine.Locus_7495
6	2	96.9	i32865_1404-HRM	TAGTCAGAATCTTCCTCTCCTGGT	AGTGGAGGAAGATGAAGAAGTTGA	A	G	2	193.2	AC.Combine.Locus_19254
7	2	100.7	i32416_685-HRM	AGCAATGAAGTACGATTTACAGCA	TGAAGAAGAACCCTCCAACGTTAT	T	C	2	203.3	AC.Combine.Locus_17664
8	2	116.5	i33538_1298-HRM	AATCGCCATTAGAAAGCTTTACCG	TACACTAAACCCTACAAACGTCGA	C	G	2	217.9	AC.Combine.Locus_250
9	2	126.2	i26131_2020-HRM	GCTTCTTTGGCCCCATATTCAAG	CATTTGCATAATGTGAGAAAGCGC	T	C	2	227.0	AC.Combine.Locus_1772
10	3	0	i35099_237-HRM	GAAGGATGCTGGTAAGAGGTCTAC	ATTATCCAAACCTGTACCCGTGAA	C	T	3	0.0	AC.Combine.Locus_31460
11	3	20.2	i39498_201-HRM	AAGAGTTGGGTGTGAAAGGAGATT	CCTGTGTTGAGATTTGGGGATTTC	T	C	3	12.7	AC.Combine.Locus_89589
12	3	25.8	i33531_1155-HRM	CCTTATGCAGATTCACCATGGAAG	CGGATCTCGTTTAACAGTGGAAAG	T	C	3	12.7	AC.Combine.Locus_14239
13	3	39.2	i35038_601-HRM	GACTTGGAGTGCAGTTGAGAC	AATCATCGGGCCTCAATGTTCAA	G	A	3	25.1	AC.Combine.Locus_14173
14	3	100.9	i26005_1583-HRM	CAGAGATCTCAACTTGTTCCCTGA	ATTGCATACCTCGAATCGCCTTTA	A	G	3	174.0	AC.Combine.Locus_9082
15	4	0	i29163_2080-HRM	TTCAGTAAACAAAAGATCGGCTGA	AAATCGGCCATCTTATTGTCTCCA	G	A	4B	0.0	AC.Combine.Locus_8343
16	4	26.2	i26442_1225-HRM	ACATTCTTCAAAGCGGTAACAACC	CAGTCATATACACCTTTATGCAAGT	A	G	4A	28.3	AC.Combine.Locus_1490
17	4	54.3	i26526_748-HRM	AGGAGGTAATGCACTGATTATTTGT	TGCACAATTGAGAGAAGGTGTTTT	A	G	4B	52.4	AC.Combine.Locus_10803
18	4	62.0	i32123_1465-HRM	CACGAATCCATAAGAGTTATCGCA	TGATCAGGGCTAGGAAAGTTTGAT	T	C	4B	52.4	AC.Combine.Locus_5789
19	5	68.6	i25881_1343-2-HRM	TTCTGACAATTTGACCGGTTGAAG	CGCGGTTACTCAAGGTTTAAGATT	T	C	5	59.0	AC.Combine.Locus_3681
20	5	69.1	i25881_1343-1-HRM	CCATCCTGAACACGATAAACCTTC	GATTAGGAGTTTGGCTTTGCTGTG	C	T	5	59.0	AC.Combine.Locus_3681
21	5	85.6	i29728_1131-HRM	CACAAAGGGGAATCAATAATCGCA	GCCTGCTCTTGGAACTGATAAAAT	T	C	5	119.4	AC.Combine.Locus_2597
22	5	112.1	i30593_868-HRM	TAAAGACCACAACAGACTCGTTCA	TTGGTTAAGGGAGTCTATGTGAGC	T	C	5	178.6	AC.Combine.Locus_70708
23	5	115.2	i36364_683-HRM	GAACCCGCCTAAGAACCAGAA	TTCATCCTCGGACTGTCTACTAGA	G	A	5	176.4	AC.Combine.Locus_24909
24	5	118.4	i29592_700-HRM	CTTCTAGAGTTGGTGTTGTGTCCA	ACTCTATGCAAACTTCACCTGAGA	G	C	5	178.6	AC.Combine.Locus_4560
25	6	54.8	i30880_1388-HRM	CGTTGGAAGATTATGTTCATCGCA	TTGGCTGCAGTGAAGTAGGTATAG	C	A	6B	9.3	AC.Combine.Locus_8405
26	6	73.3	i35768_1013-HRM	GACATGCCGCAATCCAAGATTAG	CGGTAGATGGTGAAATTTGTGTCA	T	C	6B	30.2	AC.Combine.Locus_37095
27	6	91.1	i32739_152-HRM	AAACGGCCATCTTGAAGCAATAGA	GCAAAACTTGGTCAGATAGAGAGC	G	A	6B	41.0	AC.Combine.Locus_12004
28	6	91.7	i36782_698-HRM	GCATGTTGATAGGAATTCGAATGC	GTGTTGTCTTGTTCTCGTGGTTC	A	T	6B	44.3	AC.Combine.Locus_15991
29	7	15.6	i39918_357-HRM	ATAACCTCTTCTCAATTCGAACTTC	TCCGATCCTCAATGACGACAATAA	C	G	7	39.1	AC.Combine.Locus_48105
30	7	38.8	i29101_1894-HRM	CATACCAACCTGCACACTTAAACA	GTACCATAGCGACATCCTATAGCC	A	G	7	92.6	AC.Combine.Locus_854
31	7	43.4	i28923_2628-HRM	TACTATGGGAATTAGCTACGATGC	AACCGTCTATCCTGGAACCCTA	C	T	7	94.8	AC.Combine.Locus_51
32	8	18.2	i31126_1315-HRM	ACTCTACTTGATGTTCAGTGTGGC	CTTGTCATCATCTTTCCCTAGGCT	T	C	8	18.2	AC.Combine.Locus_2785
33	8	22.0	i30907_420-HRM	TGGCTCTACTGGGGATTTGTTAAA	CACTCGGCAAATATCCCTGGTAG	C	T	8	15.4	AC.Combine.Locus_65044
34	8	66.9	i31261_1350-HRM	GTCCCCTAGAAACAGATCTCCAAC	CGACTGTGACTTTTCGGGAATTTA	A	C	8	69.4	AC.Combine.Locus_815

^z^ Map information originated from the results of Duangjit et al. [30]. ^y^ Transcript ID information originated from the results of Kim et al. [31]. ^x^ Chr. No., chromosome number.

**Table 4:** Summary of the onion genetic linkage map constructed from an F_2_ population of SP3B×H6.

Chromosome No.	Length of Linkage Maps (cM)	Total Number of Markers (A+B)	Number of SNPs Resulting from GBS (A) ^z^	Number of HRM Markers (B) ^y^
1	122.5	36	34	2
2	127.9	53	46	7
3	134.1	42	37	5
4	94.5	38	34	4
5	142.8	45	39	6
6	111.3	64	60	4
7	73.9	38	35	3
8	74.3	37	34	3
Total	881.4	353	319	34
Average	110.2	44	40	4

^z^ GBS, genotyping-by-sequencing. ^y^ HRM, high-resolution melting.

**Table 5:** Segregation analysis of onion bulb colors in an F_2_ population of SP3B×H6.

Population	Generation	Number of Onion Plants			Expected Ratio	X^2^ Value	*p* Value
		Red Bulb	Yellow Bulb	Total			
SP3B×H6	F_2_	51	18	69	3:1	0.0435	0.835

**Table 6:** Summary of significant quantitative trait loci (QTLs) for anthocyanin synthesis and content identified in the F_2_ onion population of SP3B×H6.

Trait	QTL	Chr.	Marker Interval	QTL Peak Position (cM)	Additive Effect	Dominance Effect	*R*^2 z^ (%)	LOD ^y^ Value	LOD Threshold ^x^
Anthocyanin synthesis	*qAS7.1*	7	T25488.1_1462-i39918_357-HRM	13.8	−0.9573	0.2401	87.61	9.19	5.3
Anthocyanin content	*qAC4.1*	4	T57513.1_314-T53764.1_356	7.0	−0.0299	−0.0513	19.43	3.26	3.0
	*qAC4.2*	4	T84695.1_220-i32123_1465-HRM	62.0	−0.0399	−0.0335	26.28	3.03	3.0

^z^*R*^2^, proportion of variance explained by the QTL at the test site. ^y^ Logarithm of the odds (LOD). ^x^ LOD threshold was determined with a 1000 times permutation test.

## Supplementary Materials

The following are available online at https://www.mdpi.com/2223-7747/9/5/616/s1, Table S1: The SNP matrix generated by GBS analysis, Table S2: Statistics for GBS-based SNP and HRM markers mapped in this study, Table S3: Cosegregation of the SNP marker (T25488.1_1462) with bulb color in an F_2_ population of SP3B×H6.

## Abbreviations

AC	anthocyanin content
AFLP	amplified fragment length polymorphism
ANS	anthocyanidin synthase
AS	anthocyanin synthesis
CAPS	cleaved amplified polymorphic sequence
CIM	composite interval mapping
cM	centi Morgan
ddRAD-seq	double digest restriction site-associated DNA sequencing
DFR	dihydroflavonol 4-reductase
EST	expressed sequence tag
GBS	genotyping-by-sequencing
HRM	high-resolution melting
KASP	kompetitive allele specific PCR
LOD	logarithm of odds
MAS	marker-assisted selection
NGS	next-generation sequencing
QTL	quantitative trait loci
RAPD	random amplified polymorphic DNA
RFLP	restriction fragment length polymorphism
SNP	single-nucleotide polymorphism
SSCP	single-strand conformation polymorphism
SSR	simple sequence repeat
